# Xanthine oxidase and aldehyde oxidase contribute to allopurinol metabolism in rats

**DOI:** 10.1186/s40780-022-00262-x

**Published:** 2022-12-08

**Authors:** Yoshitaka Tayama, Kazumi Sugihara, Seigo Sanoh, Katsushi Miyake, Shigeyuki Kitamura, Shigeru Ohta

**Affiliations:** 1 Faculty of Pharmaceutical Science, Hiroshima International University, 5-1-1 Hirokoshingai, Kure-shi, Hiroshima, 737-0112 Japan; 2 School of Pharmaceutical Health Sciences, Wakayama Medical University, 25-1 Shichibancho, Wakayama, 640-8156 Japan; 3 Nihon Pharmaceutical University, Komuro 10281, Inamachi, Kitaadachi-gun, Saitama, 362-0806 Japan

**Keywords:** Aldehyde oxidase, Xanthine oxidase, Allopurinol, Oxypurinol, Strain differences

## Abstract

**Background:**

Allopurinol is used to treat hyperuricemia and gout. It is metabolized to oxypurinol by xanthine oxidase (XO), and aldehyde oxidase (AO). Allopurinol and oxypurinol are potent XO inhibitors that reduce the plasma uric acid levels. Although oxypurinol levels show large inter-individual variations, high concentrations of oxypurinol can cause various adverse effects. Therefore, it is important to understand allopurinol metabolism by XO and AO. In this study we aimed to estimate the role of AO and XO in allopurinol metabolism by pre-administering Crl:CD and Jcl:SD rats, which have known strain differences in AO activity, with XO inhibitor febuxostat.

**Methods:**

Allopurinol (30 or 100 mg/kg) was administered to Crl:CD and Jcl:SD rats with low and high AO activity, respectively, after pretreatment with or without febuxostat. The serum concentrations of allopurinol and oxypurinol were measured, and the area under the concentration-time curve (AUC) was calculated from the 48 h serum concentration-time profile. In vivo metabolic activity was measured as the ratio AUC_oxypurinol_ /AUC_allopurinol_.

**Results:**

Although no strain-specific differences were observed in the AUC_oxypurinol_/AUC_allopurinol_ ratio in the allopurinol (30 mg/kg)-treated group, the ratio in Jcl:SD rats was higher than that in Crl:CD rats after febuxostat pretreatment. Contrastingly, the AUC ratio of allopurinol (100 mg/kg) was approximately 2-fold higher in Jcl:SD rats than that in Crl:CD rats. These findings showed that Jcl:SD rats had higher intrinsic AO activity than Crl:CD rats did.

However, febuxostat pretreatment substantially decreased the activity, as measured by the AUC ratio using allopurinol (100 mg/kg), to 46 and 63% in Crl:CD rats and Jcl:SD rats, respectively, compared to the control group without febuxostat pretreatment.

**Conclusions:**

We elucidated the role of XO and AO in allopurinol metabolism in Crl:CD and Jcl:SD rats. Notably, AO can exert a proportionately greater impact on allopurinol metabolism at high allopurinol concentrations. AO’s impact on allopurinol metabolism is meaningful enough that individual differences in AO may explain allopurinol toxicity events. Considering the inter-individual differences in AO activity, these findings can aid to dose adjustment of allopurinol to avoid potential adverse effects.

**Supplementary Information:**

The online version contains supplementary material available at 10.1186/s40780-022-00262-x.

## Background

Allopurinol is widely used to treat hyperuricemia and gout, uric acid stones in the kidney, and acute uric acid nephropathy during chemotherapy for malignancies (tumor lysis syndrome). Allopurinol is metabolized to oxypurinol by both xanthine oxidase (XO) and aldehyde oxidase (AO). Oxypurinol is primarily eliminated by the kidneys. Both allopurinol and oxypurinol are competitive inhibitors of XO, which inhibit the conversion of hypoxanthine to xanthine and xanthine to uric acid, resulting in reduced blood uric acid levels. The potencies of oxypurinol and allopurinol for XO inhibition is similar, but the half-life (13–18 h) of oxypurinol is much longer than that of allopurinol (0.6–1.6 h) in humans. Therefore, the in vivo inhibitory effect of allopurinol on XO is primarily attributed to oxypurinol [1].

Okada et al. [2] reported a relationship between serum oxypurinol concentration adjusted for the allopurinol dose and creatinine clearance in 89 Japanese patients. Large inter-individual differences in serum oxypurinol concentration (approximately 4-fold) were observed despite almost the same value of creatinine clearance. Similar results were reported by Stamp et al. [3].

The optimal concentration range of oxypurinol needed to regulate serum urate is 30–100 μM [4]. High levels of oxypurinol (> 100 μM) tend to cause various adverse effects, such as bone marrow depression and exfoliative dermatitis [5]. Therefore, the conversion of allopurinol to oxypurinol should be monitored and predicted in the liver, considering inter-individual differences in order to prevent adverse effects.

The involvement of XO and AO on allopurinol metabolism has been evaluated through in vitro or in silico studies [6, 7]. Moriwaki et al. [8] reported that each Km value of allopurinol for XO and AO in the in vitro metabolic study was 0.5 mM and 1.1 mM, respectively.

The relative contributions of XO and AO to allopurinol metabolism in vivo have not yet been fully established. To the best of our knowledge, only one in vivo study reported that AO played a minor role in allopurinol metabolism through a metabolic study using an XO-specific inhibitor [1]. However, the relative contributions of XO and AO to allopurinol metabolism in vivo remain unclear.

XO and AO are cytosolic enzymes that contain flavin adenine dinucleotide (FAD), molybdenum atoms, and iron-sulfur centers. They have a very close evolutionary relationship and show a high degree of amino acid sequence homology [9, 10]. AO activity, particularly, has marked strain differences in mice and rats [11]. Among them, Jcl:SD rats show higher AO activity than Crl:CD rats do [12]. Contrastingly, in humans, the individual differences in the in vivo AO activity levels can range up to four-fold [13]. Thus, these differences in serum oxypurinol concentrations may depend on differences in AO activity.

This study aimed to evaluate the relative contributions of AO and XO to the in vivo metabolism of allopurinol, considering the strain differences in intrinsic AO activity between Crl:CD and Jcl:SD rats. These findings may be important in explaining individual differences in serum oxypurinol concentrations, which may be related to both therapeutic and adverse effects, and are expected to contribute to optimal allopurinol treatment.

## Methods

### Materials and chemicals

*N*^1^-methylnicotinamide (NMN) and *N*′-methylnicotinamide were obtained from Tokyo Chemical Industry Co., Ltd. (Tokyo, Japan). *N*^1^-Methyl-2-pyridone-5-carboxamide (2-PY) and *N*^1^-methyl-4-pyridone-3-carboxamide (4-PY) were prepared according to the methods described by Pullman and Colowick [14] and Shibata et al. [15], respectively. Oxypurinol, 1-methyluric acid, 1-methylxanthine, and acetaminophen were purchased from Sigma-Aldrich Japan Co., Ltd. (Tokyo, Japan). Febuxostat [2-(3-cyano-4-isobutoxyphenyl)-4-methyl 1,3-thiazole-5-carboxylic acid], a selective and potent inhibitor of XO [16], was procured from LKT Laboratories, Inc. (Minnesota, USA). Allopurinol and caffeine were purchased from Nacalai Tesque Co. Ltd. (Kyoto, Japan). Sodium carboxymethyl cellulose was purchased from Maruishi Pharmaceutical Co. Ltd. (Osaka, Japan).

### Animals

Jcl:SD and Crl:CD rats (10 weeks of age, male) were obtained from Clea Japan Co. Ltd. (Tokyo, Japan) and Charles River Laboratories Japan Inc. (Yokohama, Japan), respectively. The animals were housed in cages at 22 ± 2 °C and 55 ± 5% relative humidity with a 12-h light/dark cycle, free access to tap water, and a standard pellet diet CE-2 (Clea Japan Co., Ltd., Tokyo, Japan). The animal protocol was approved by the Animal Care and Use Committee of Hiroshima International University (AE14–007).

First, we confirmed the intrinsic XO and AO activities in Crl:CD and Jcl:SD rats by using the caffeine test and the ratio of pyridines before allopurinol pharmacokinetic (PK) analysis [12, 17]. After the allopurinol PK analysis, we confirmed the intrinsic XO and AO activities under the effects of febuxostat using the same method.

### Assessment of in vivo intrinsic XO activity

XO activity was estimated using caffeine as a probe compound, which was metabolized to 1-methylxanthine (1-MX) and then to 1-methyl uric acid (1-MU) by XO. The molar ratio of urinary excretion of 1-MU acid to 1-MX reflects in vivo intrinsic XO activity (caffeine test) [17]. First, the rats were administered febuxostat (10 mg/kg) or 1% sodium carboxymethyl cellulose solution, followed by caffeine (30 mg/kg). Urine was collected for 24 h. To determine the concentrations of 1-MU and 1-MX, urine was pretreated as described by Grant et al. [18]. Water (0.3 mL), 0.4 mg acetaminophen (internal standard), and 0.6 g of ammonium sulfate were added to the urine sample (0.5 mL). The mixture was briefly vortexed. Dichloromethane/isopropanol (5 mL; 85:15, v/v) was added to the mixture and then vigorously vortexed for 30 s. Subsequently, the mixture was centrifuged at 900 x g for 10 min. The organic phase was then removed and evaporated. The residue (20 μL) was resuspended in the HPLC mobile phase and subjected to HPLC on a Mightysil RP-18 (4.6 mm × 150 mm) column (Kanto Chemical Co., Inc., Tokyo, Japan) with ultraviolet detection at 280 nm. The mobile phase consisted of a mixture of 0.5% acetic acid/methanol (92:8, v/v), with a flow rate of 0.5 mL/min. The elution times of 1-MU, 1-MX, and acetaminophen (internal standard) were 15.0, 19.6, and 23.0 min, respectively.

### Assessment of in vivo intrinsic AO activity

AO activity was estimated according to the pyridine ratio method reported by Sugihara et al. [12]. NMN, which is synthesized from nicotinamide by nicotinamide methyltransferase, is widely distributed in animals and metabolized by AO to 2-PY and 4-PY. The ratio of 2-PY + 4-PY to NMN + 2-PY + 4-PY (the ratio of pyridines: RP) in urine can be used to estimate the in vivo intrinsic AO activity.

Urine was collected for 12 h. To measure 2-PY and 4-PY, a 0.1 mL aliquot of urine was added to 0.4 mL water, 10 μg *N*′-methylnicotinamide (an internal standard) and 0.6 g potassium carbonate. The mixture was extracted three times using 5 mL of diethyl ether, and the extract was evaporated to dryness. The residue was again dissolved in 0.1 mL methanol and subjected to HPLC on a Capcell Pak C18 UG120 column (25 cm × 4.6 mm, Shiseido Co. Ltd., Tokyo, Japan). The mobile phase was acetonitrile-water (3:97, v/v), and the flow rate was 0.5 mL/min. The detection wavelength was set at 254 nm. The elution times of 4-PY, 2-PY, and *N*′-methylnicotinamide (an internal standard) were 13.5, 14.7 and 30.0 min, respectively.

To measure NMN levels, urine (0.1 mL) was added to a mixture of 1 mL of 0.2 M isonicotinamide and 0.5 mL of 0.1 M acetophenone on ice. One milliliter of 6 M sodium hydroxide and 0.5 mL of formic acid were added. After allowing to stand on ice for 10 min the mixture was boiled in a water bath for 5 min. The supernatant was collected and subjected to HPLC on a LiChrospher Select B column (15 cm × 4.6 mm, Merck Co. Ltd., Tokyo, Japan). The mobile phase was acetonitrile /0.1 M potassium dihydrogen phosphate (1:4, v/v) and the flow rate was 0.5 mL/min. The fluorometric detector was set at the excitation and emission wavelengths of 382 and 440 nm, respectively. The elution time of 1-methyl-7-phenyl-15-dehydro-5-oxo-1,6-naphthyridine, a derivative of NMN, was 6.2 min.

### Allopurinol PK analysis

**Fig. 1:**
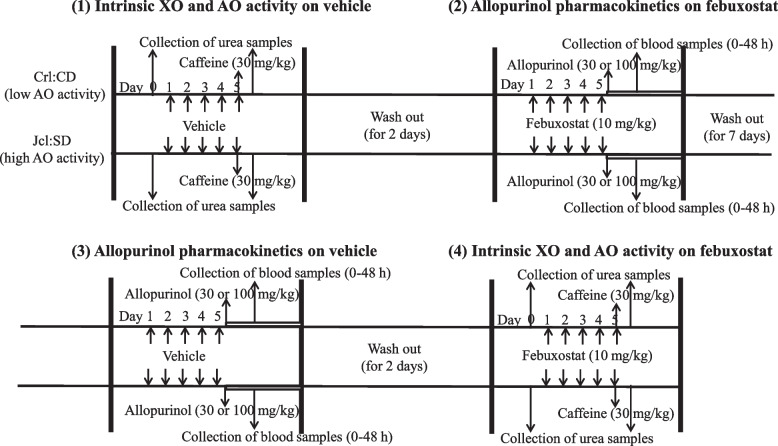
The experimental groups and treatment schedules. (1) Intrinsic XO and AO activities on vehicle. Crl:CD (low AO activity) and Jcl:SD rats (high AO activity) were orally administered vehicle once daily for 5 days. Urea samples were collected twice. One sample was collected before the administration of the vehicle to assess intrinsic AO activity. The other was collected after the administration of the vehicle and caffeine for the assessment of intrinsic XO activity. (2) Allopurinol pharmacokinetics on Febuxostat. Crl:CD rats (low AO activity) and Jcl:SD rats (high AO activity) were orally administered febuxostat (10 mg/kg) once daily for 5 days. Allopurinol (30 or 100 mg/kg) was administered orally. Blood samples were collected 0, 1, 2, 4, 8, 24, and 48 h after allopurinol administration. (3) Allopurinol pharmacokinetics on vehicle. Crl:CD (low AO activity) and Jcl:SD rats (high AO activity) were orally administered vehicle once daily for 5 days. Allopurinol (30 or 100 mg/kg) was orally administered. Blood samples were collected 0, 1, 2, 4, 8, 24, and 48 h after allopurinol administration. (4) Intrinsic XO and AO activities on febuxostat. Crl:CD (low AO activity) and Jcl:SD rats (high AO activity) were orally administered febuxostat once daily for 5 days. Urea samples were collected twice. One sample was collected before the administration of febuxostat to assess intrinsic AO activity. The other was collected after the administration of febuxostat and caffeine for the assessment of intrinsic XO activity

Febuxostat (10 mg/kg body weight suspended in 1% sodium carboxymethyl cellulose) was orally administered to rats of each strain (11 weeks of age, male) once per day for 5 days. One hour after the final administration of febuxostat, allopurinol was orally administered to rats at a single dose of 30 or 100 mg/kg body weight suspended in 1% sodium carboxymethyl cellulose. Blood samples (approximately 0.1 mL) were collected from the tail veins of rats at 0, 1, 2, 4, 8, 24, and 48 h after allopurinol administration. Serum samples were obtained via centrifugation. After a washout period (7 days), rats were orally administered 1% carmellose sodium solution, instead of febuxostat, once daily for 5 days (vehicle group), followed by allopurinol administration. This was followed by a PK analysis using the methods described above. A schematic presentation of the treatment schedule and groups is shown in Fig. 1.

### Assay of the serum concentrations of allopurinol and oxypurinol

Equal volumes of serum and water (0.05 mL each) were mixed. This mixture was vortexed, and 1 M perchloric acid (0.1 mL) was added to precipitate the proteins. After centrifugation, 10 μL of supernatant was injected into the HPLC system. The mobile phase consisted of a mixture of acetonitrile and 10 mM phosphate buffer (pH 3.5) (8:1000, v/v). The flow rate was 0.5 mL/min at 40 °C. UV detection was performed at a wavelength of 254 nm. The elution times of allopurinol and oxypurinol were 17.7 min and 15.8 min, respectively.

### Statistical analysis

Data are presented as mean ± standard deviation (S.D.). The statistically significant difference between two groups, evaluated using the Student’s *t*-test, was considered at *p* < 0.05.

## Results

### In vivo intrinsic XO activity in Crl:CD and Jcl:SD rats and the effect of febuxostat

**Fig. 2:**
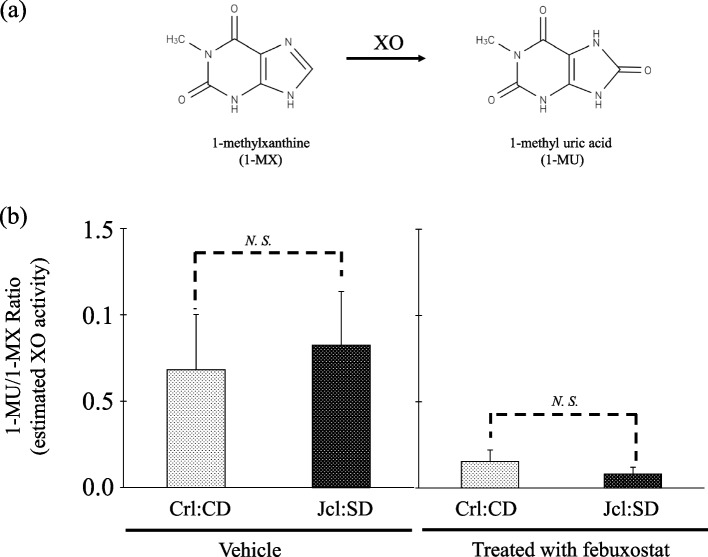
**a** Metabolic pathways of 1-methylxanthine (1-MX) by xanthine oxidase(XO), and (**b**) Effect of febuxostat on xanthine oxidase (XO) activities in Crl:CD and Jcl:SD rats. Xanthine oxidase (XO) activity was estimated as the ratio of 1-methyl uric acid (1-MU) to 1-methylxanthine (1-MX). Each bar represents the mean ± S.D. recorded from six experiments. ** *p* < 0.01; *N.S.* not significant

We evaluated in vivo intrinsic XO activity, with or without febuxostat, by measuring the ratio of 1-MU to 1-MX in urine after administration of caffeine in Crl:CD and Jcl:SD rats. In the vehicle group, the 1-MU/1-MX ratio (mean ± S.D.) was 0.682 ± 0.103 and 0.825 ± 0.096 in Crl:CD and Jcl:SD rats, respectively. The difference in the 1-MU/1-MX ratio between the two strains was not statistically significant. In addition, after febuxostat administration, these values were 0.153 ± 0.005 and 0.082 ± 0.001 in Crl:CD and Jcl:SD rats, respectively. No statistically significant differences in the 1-MU/1-MX ratio was detected between the two strains after febuxostat administration (Fig. 2).

### In vivo intrinsic AO activity in Crl:CD and Jcl:SD rats and the effect of febuxostat

**Fig. 3:**
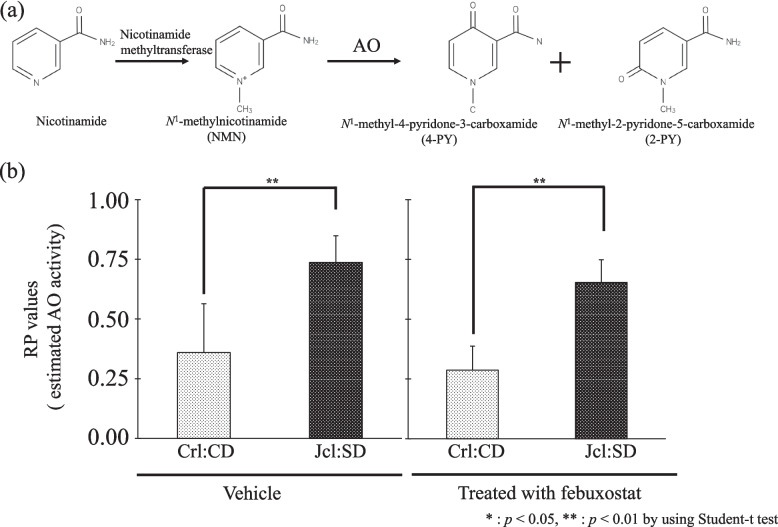
**a** Metabolic pathways of *N*-1-methylnicotinamide by aldehyde oxidase (AO), and (**b**) Effect of febuxostat on aldehyde oxidase (AO) activity in Crl:CD and Jcl:SD rats. Aldehyde oxidase (AO) activity was estimated as the RP value. Each bar represents the mean ± S.D. of six experiments. ** *p* < 0.01; *N.S.* not significant

The in vivo intrinsic AO activity was estimated from the RP values. In the vehicle group, the RP value (mean ± S.D.) of Jcl:SD rats (0.737 ± 0.013) was approximately two-fold higher than that of the Crl:CD rats (0.359 ± 0.042) (*p* < 0.01, Fig. 3). Furthermore, the RP values were 0.287 ± 0.010 and 0.654 ± 0.009 in Crl:CD and Jcl:SD rats, respectively, after febuxostat administration. The RP value of Jcl:SD rats was approximately two-fold higher than that of Crl:CD rats. (*p* < 0.01, Fig. 3).

### Serum concentration-time profiles of allopurinol and oxypurinol

**Fig. 4:**
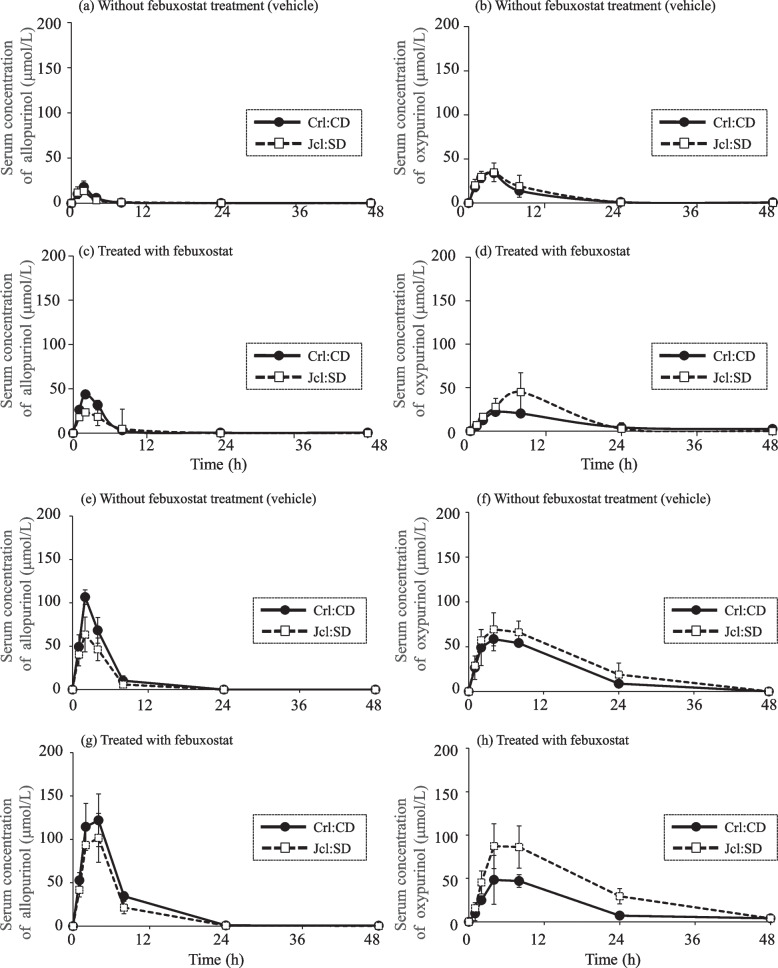
Time profiles of serum concentration of allopurinol and oxypurinol. Crl:CD and Jcl:SD rats were administered allopurinol (30 mg/kg): **a-d** or (100 mg/kg): **e**-**h**. Each point represents the mean ± standard deviation (SD) of three experiments. **a** Serum allopurinol concentration in vehicle-treated rats. **b** Serum oxypurinol concentration in vehicle-treated rats. **c** Serum concentration of allopurinol in rats treated with febuxostat (10 mg/kg) for 5 days. **d** Serum concentration of oxypurinol in rats treated with febuxostat (10 mg/kg) for 5 days. **e** Serum concentrations of allopurinol in vehicle-treated rats. **f** Serum oxypurinol concentrations in vehicle-treated rats. **g** Serum concentrations of allopurinol in rats treated with febuxostat (10 mg/kg) for 5 days. **h** Serum concentration of oxypurinol in rats treated with febuxostat (10 mg/kg) for 5 days

Allopurinol (30 or 100 mg/kg) was orally administered to rats pretreated with vehicle or febuxostat (10 mg/kg) once daily for 5 days. Serum samples were collected at 0, 1, 2, 4, 8, 24, and 48 h to measure serum concentrations of allopurinol and oxypurinol (Fig. 4).

In vehicle-pretreated rats administered with allopurinol (30 mg/kg), the concentration profiles of allopurinol and oxypurinol showed no strain differences (Fig. 4a, b). However, febuxostat-pretreated Crl:CD rats initially showed higher concentrations of allopurinol than Jcl:SD rats (2 and 4 h, Fig. 4c), and subsequently showed lower concentrations of oxypurinol than Jcl:SD rats (8 h, Fig. 4d).

On allopurinol administration (100 mg/kg), vehicle-treated Crl:CD rats showed higher concentrations of allopurinol (Fig. 4e) and lower concentrations of oxypurinol than the Jcl:SD rats (Fig. 4f). Similarly, febuxostat-treated Crl:CD rats showed higher concentrations of allopurinol (Fig. 4g) and lower concentrations of oxypurinol than that found in Jcl: SD rats (Fig. 4h).

### AUC of allopurinol and its metabolite oxypurinol with or without febuxostat

The area under the concentration-time curve (AUC_allopurinol_) was calculated from the 48 h serum concentration-time profile of allopurinol. Similarly, the AUC_oxypurinol_ was obtained from oxypurinol.

The obtained values of AUC_allopurinol_, AUC_oxypurinol_ and AUC_total_ (AUC_allopurinol_ + AUC_oxypurinol_) are shown in Table 1. The AUC_allopurinol_ in case of allopurinol (100 mg/kg) increased 4- to 7-fold, the AUC_oxypurinol_ increased 2- to 4-fold, and the AUC_total_ increased 3- to 4-fold higher than those at 30 mg/kg allopurinol with or without febuxostat.

**Table 1:**
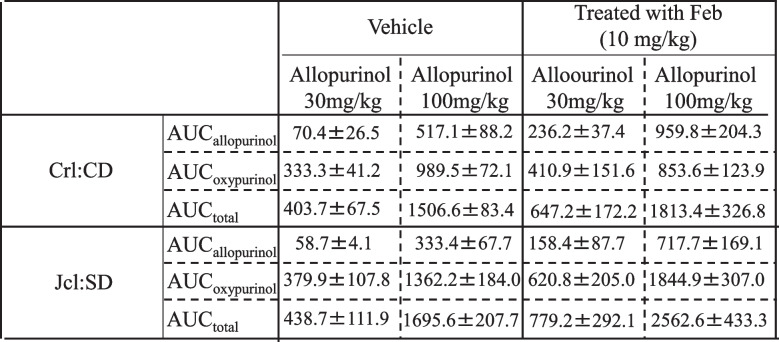
Values of AUCallopurinol, AUCoxypurinol, and AUCtotal in Crl:CD and Jcl:SD rats. Rats were administered allopurinol 30 or 100 mg/kg with or without febuxostat (10 mg/kg). AUCtotal is the sum of AUCallopurinol and AUCoxypurinol. Each bar represents the mean ± S.D. of 3 experiments

Means±S. D. (mmol・h/L)

**Fig. 5:**
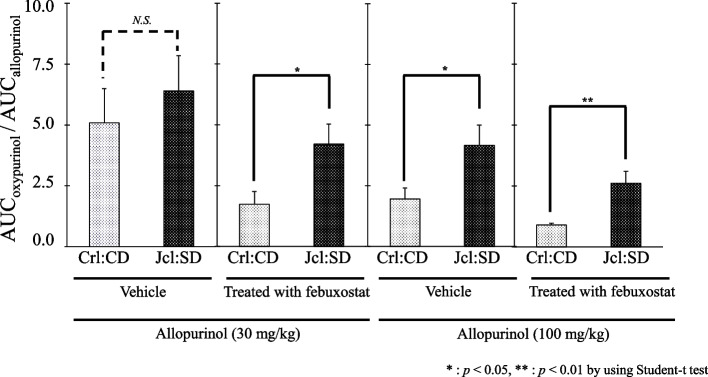
The AUC_oxypurinol_/AUC_allopurinol_ ratio in different treatment groups. Crl:CD and Jcl:SD rats were administered allopurinol 30 or 100 mg/kg with or without febuxostat (10 mg/kg). Each bar represents the mean ± standard deviation (SD) of three experiments. ** *p* < 0.01; *N.S.* not significant

We calculated the ratio of AUC_oxypurinol_ to AUC_allopurinol_ to estimate the in vivo metabolic activity of the conversion of allopurinol to oxypurinol (Fig. 5).

At 30 mg/kg allopurinol, vehicle-treated rats showed no significant differences in the ratio AUC_oxypurinol_/AUC_allopurinol_ in either Crl:CD or Jcl:SD rats. Upon febuxostat administration, each of AUC_oxypurinol_ and AUC_allopurinol_ reduced to 35 and 65% compared to that in the vehicle group (30 mg/kg of allopurinol alone) in Crl:CD and Jcl:SD rats, respectively. Overall, febuxostat-treated Jcl:SD rats showed a higher ratio than Crl:CD rats.

At 100 mg/kg allopurinol, the ratio AUC_oxypurinol_ /AUC_allopurinol_ in vehicle-treated Jcl:SD rats was approximately two-fold higher than that in the Crl:CD rats (*p* < 0.05). These ratios were similar to those of allopurinol (30 mg/kg)- and febuxostat-treated rats. However, upon febuxostat administration, each ratio was reduced to 46 and 63% compared to those of the vehicle groups in Crl:CD and Jcl:SD rats, respectively. Overall, the ratio AUC_oxypurinol_ /AUC_allopurinol_ in febuxostat-treated Jcl:SD rats was significantly higher than that in Crl:CD rats (*p* < 0.01).

## Discussion

According to the 2012 American College of Rheumatology Guidelines, allopurinol and febuxostat were considered as the first-line of treatment for hyperuricemia and gout. However, febuxostat has been associated with a higher risk of cardiovascular disease-related mortality and higher costs compared to allopurinol. Therefore, the 2020 American College of Rheumatology Guidelines recommend allopurinol for many patients [19].

Allopurinol is rapidly metabolized to oxypurinol by XO and AO [1, 6, 7], both of which are active XO inhibitors. However, oxypurinol shows large inter-individual differences in plasma concentrations after allopurinol administration [20]. Nonetheless, high concentrations of oxypurinol can cause myelosuppression and exfoliative dermatitis [5]. Therefore, considering these side effects, the degree of inter-individual differences should be noted.

Inter**-**individual difference in XO activity in humans is only 1.7-times [21], whereas inter-individual differences in AO activity up to 4-fold are known to occur in Japanese adults [13]. AO metabolic activity is known for its individual differences; thus, it is important to understand the roles of XO and AO activities to allopurinol metabolism.

In this study, Crl:CD and Jcl:SD rats, which show differences in AO intrinsic activity, were examined. Moreover, based on the metabolic index (RP ratio) of NMN by AO, we confirmed that AO activity in Jcl:SD rats is higher than that in Crl:CD rats (Fig. 3). Altogether, we found no differences in XO activity between these two rat strains according to the metabolic index (caffeine test) of 1-MX by XO (Fig. 2). Furthermore, we *used* febuxostat, a known XO inhibitor, to investigate the contribution of AO and XO to allopurinol metabolism. It can be hypothesized that febuxostat inhibited XO activity, but not AO activity, considering the results shown in Figs. 2 and 3.

Weidert et al. [22] reported that febuxostat is a poor inhibitor of AO activity in human cytosol. We evaluated the effect of febuxostat on in vitro XO and AO activities in the liver cytosol of Jcl:SD rats (Supplemental Fig. 1). XO activity decreased with increasing febuxostat dose. However, AO activity was not affected by febuxostat at the examined concentration range.

In Fig. 5, the AUC_oxypurinol_/AUC_allopurinol_ ratio in the allopurinol (30 mg/kg) group without febuxostat (vehicle) treatment was slightly higher for Jcl:SD than for Crl:CD; however, the difference was not statistically significant. Given the profiles of AO and XO activity in the two strains shown in Figs. 2 and 3, allopurinol (30 mg/kg) did not quantitatively reflect strain-specific variation in intrinsic AO, suggesting that a greater contribution from XO-induced metabolism may be implicated.

Febuxostat inhibited XO activity but not AO activity in vitro (Supplemental Fig. 1). The AUC ratio obtained after febuxostat treatment may be considered predominantly due to AO activity (Fig. 5). Moreover, the residual AUC ratio was higher in the Jcl:SD rats than in the Crl:CD rats. These findings reflect the originally higher AO activity in Jcl:SD rats, as shown in Fig. 3.

After administration of allopurinol (100 mg/kg) without febuxostat, AUC_allopurinol_ was approximately 6- to 7-fold higher than those of allopurinol (30 mg/kg) (Table 1). The PK evaluation of allopurinol administered at 100 mg/kg also confirmed that comparative evaluation can be carried out under conditions that are not saturated in absorption.

The AUC ratio at allopurinol (100 mg/kg) without febuxostat was approximately 2-fold higher in Jcl:SD rats than that in Crl:CD rats. These findings quantitatively reflected the higher intrinsic AO activity in Jcl:SD rats compared to that in Crl:CD rats (Fig. 3), suggesting that, with 100 mg/kg allopurinol, unlike with its lower concentration (30 mg/kg), AO’s contribution to metabolism may be greater. Moriwaki et al. [8] reported the Km value of XO for allopurinol was about half of that observed for AO. Based on this report, the high dose (100 mg/kg) may reflect the possibility of XO activity tending to saturate.

The AUC ratio at 100 mg/kg of allopurinol without febuxostat was comparable to the AUC ratio of allopurinol (30 mg/kg) combined with febuxostat. Allopurinol is competitive inhibitor of XO at low concentrations, but inhibits it noncompetitively at high concentrations. Oxypurinol, the metabolite of allopurinol, is also a non-competitive inhibitor of XO [23]. Therefore, the possibility that allopurinol (100 mg/kg) and its metabolite, oxypurinol, may have the same potential for inhibiting XO as that of febuxostat (10 mg/kg) should be considered.

Allopurinol (100 mg/kg) in febuxostat pretreated rats substantially decreased the AUC ratio to 46 and 63% in Crl:CD and Jcl:SD rats, respectively, based on the assumption that XO is completely inactivated with 100 mg Allopurinol and febuxostat treatment (Supplemental Fig. 2). Considering the inhibition of XO by febuxostat and the possibility of XO inhibition by allopurinol and oxypurinol, these ratios can be considered the metabolic contribution of AO. Altogether, the contribution of AO activity to allopurinol metabolism was estimated to be half or more when a high dose (100 mg/kg) of allopurinol was administered. Our findings also support the report that XO can be substituted by AO during the conversion of allopurinol to oxypurinol in patients with XO deficiency [24].

The serum concentration range of allopurinol used in this study was similar to the clinically effective dose [25]. To avoid adverse events, the recommended concentration of oxypurinol in humans is < 100 μM [5]. The maximum serum concentration of oxypurinol in the allopurinol (100 mg/kg)-treated group, which showed significant metabolic involvement of AO in this study, was approximately consistent with its concentration reported in humans [26]. Therefore, the doses of allopurinol (30 and 100 mg/kg), used in this study, may be suitable to evaluate the roles of AO and XO in allopurinol metabolism in the clinical context.

We studied the contribution XO and AO in rats. There are some species-specific differences in XO and AO activities. Rats have about higher XO activity (50-folds) for xanthine oxidation compared to that in humans [27]. Furthermore, compared to humans, rats tend to show lower AO activities [28]. Hence, as AO significantly contributes to allopurinol metabolism even in rats with higher XO activity and lower AO activity, humans with lower XO and higher AO activity may have an increased contribution of AO.

Furthermore, an upto 4-fold inter-individual difference of oxypurinol was detected in the human serum [2, 3]. The results of this study indicates the possibility that individual differences in AO activity are involved in allopurinol PK. In other words, individual differences in AO activity should be considered when predicting adverse effects.

Studies focusing on rat strain-specific differences in AO activity may be used to predict the effects of inter-individual differences in AO on metabolism and adverse effects in humans**.** We previously reported the assessment of inter-individual differences in AO by measuring urinary nicotinamide metabolites (RP values) as biomarkers that indicate intrinsic AO activity in humans. In the case of allopurinol administration, this can be a useful method to prevent adverse effects caused by inter-individual differences in allopurinol metabolism; because the potential of AO activity could be estimated by measurement of urinary nicotinamide metabolites (RP value). However, further clinical investigations are required.

## Conclusions

In this study, we determined the contributions of XO and AO to allopurinol metabolism. One notable observation is that AO can play a proportionately greater role in allopurinol metabolism at high allopurinol levels. Another key insight is that allopurinol toxicity events may be associated with varying levels of AO activity.

Considering the inter-individual differences in AO, these findings could contribute to optimal treatment, including dose adjustment of allopurinol to avoid potential adverse effects.

## Supplementary Information

**Additional file 1: Supplemental Fig. 1.** Effect of febuxostat on (A) XO and (B) AO activity in liver cytosol of Jcl:SD strain rats. Each bar represents the mean ± S. D. of 3 experiments. **: *p* < 0.01 by using Tukey test.

**Additional file 2: Supplemental Fig. 2.** Estimated AO contribution of allopurinol metabolism considering complete inactivation of XO with Allopurinol (100 mg) and febuxostat treatment.

## Data Availability

All data generated or analyzed during this study are included in this published article.

## Abbreviations

AO: Aldehyde oxidase
AUC: Area under the concentration-time curve
RP: Ratio of pyridines
XO: Xanthine oxidase
