# Supplementary material for: Xanthine oxidase and aldehyde oxidase contribute to allopurinol metabolism in rats
Source: J Pharm Health Care Sci. 2022 Dec 8;8:31. doi: 10.1186/s40780-022-00262-x (PMC9730672; doi:10.1186/s40780-022-00262-x)
Supplement: Supplementary file 1 — Additional file 1: Supplemental Fig. 1. Effect of febuxostat on (A) XO and (B) AO activity in liver cytosol of Jcl:SD strain rats. Each bar represents the mean ± S. D. of 3 experiments. **: p < 0.01 by using Tukey test. [file 40780_2022_262_MOESM1_ESM.pdf]

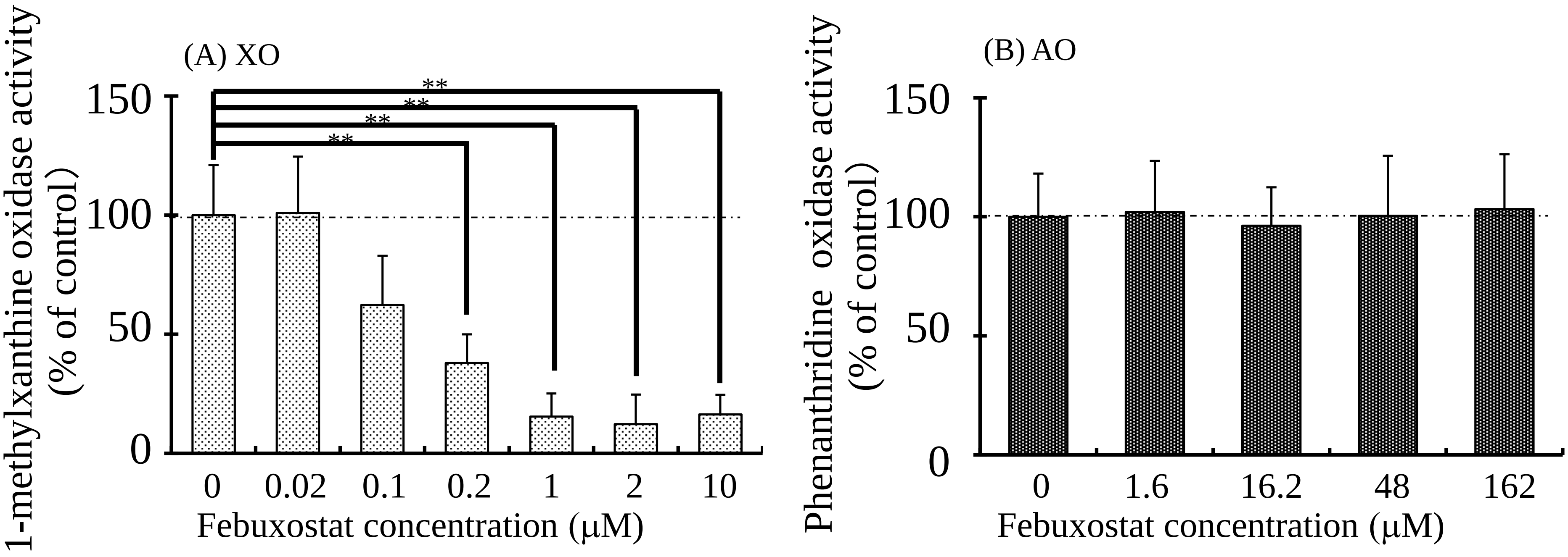

Supplemental Fig. 1 Effect of febuxostat on (A) XO and (B) AO activity in liver cytosol of Jcl:SD strain rats. Each bar represents the mean ± S. D. of 3 experiments. ** : $p < 0.01$ by using Tukey test

Livers were excised from Jcl:SD rats (6 weeks male), and the livers were homogenized. The cytosolic fraction was obtained from the homogenate livers by successive centrifugation at 9,000 x g for 20 min and 105,000 x g for 60 min. The *in vitro* XO activity was estimated by measuring the oxidation of 1-methylxanthine to 1-methyluric acid. Incubation was performed at 37 ^{o}C for 40 min. 1-Methyluric acid concentration in the mixture was determined by HPLC with ultraviolet detection at 280 nm. The *in vitro* AO activity was estimated by measuring the oxidation of phenanthridine to phenanthridone. Incubation was performed at 37 ^{o}C for 10 min. Phenanthridone concentration in the mixture was determined by HPLC with fluorometric detection (excitation at 236 nm and emission at 364 nm). For the complete methods of measuring phenanthridone concentration, refer to the report by Rashidi et al. (Rashidi MR, Amini K, Khani MY, Faridi A, Hanaee J, Sorouraddin MH. A highly sensitive RP-HPLC-fluorescence method to study aldehyde oxidase activity. J AOAC Int. 2011;94: 550-4).
