# Supplementary material for: Xanthine oxidase and aldehyde oxidase contribute to allopurinol metabolism in rats
Source: J Pharm Health Care Sci. 2022 Dec 8;8:31. doi: 10.1186/s40780-022-00262-x (PMC9730672; doi:10.1186/s40780-022-00262-x)
Supplement: Supplementary file 2 — Additional file 2: Supplemental Fig. 2. Estimated AO contribution of allopurinol metabolism considering complete inactivation of XO with Allopurinol (100 mg) and febuxostat treatment. [file 40780_2022_262_MOESM2_ESM.pdf]

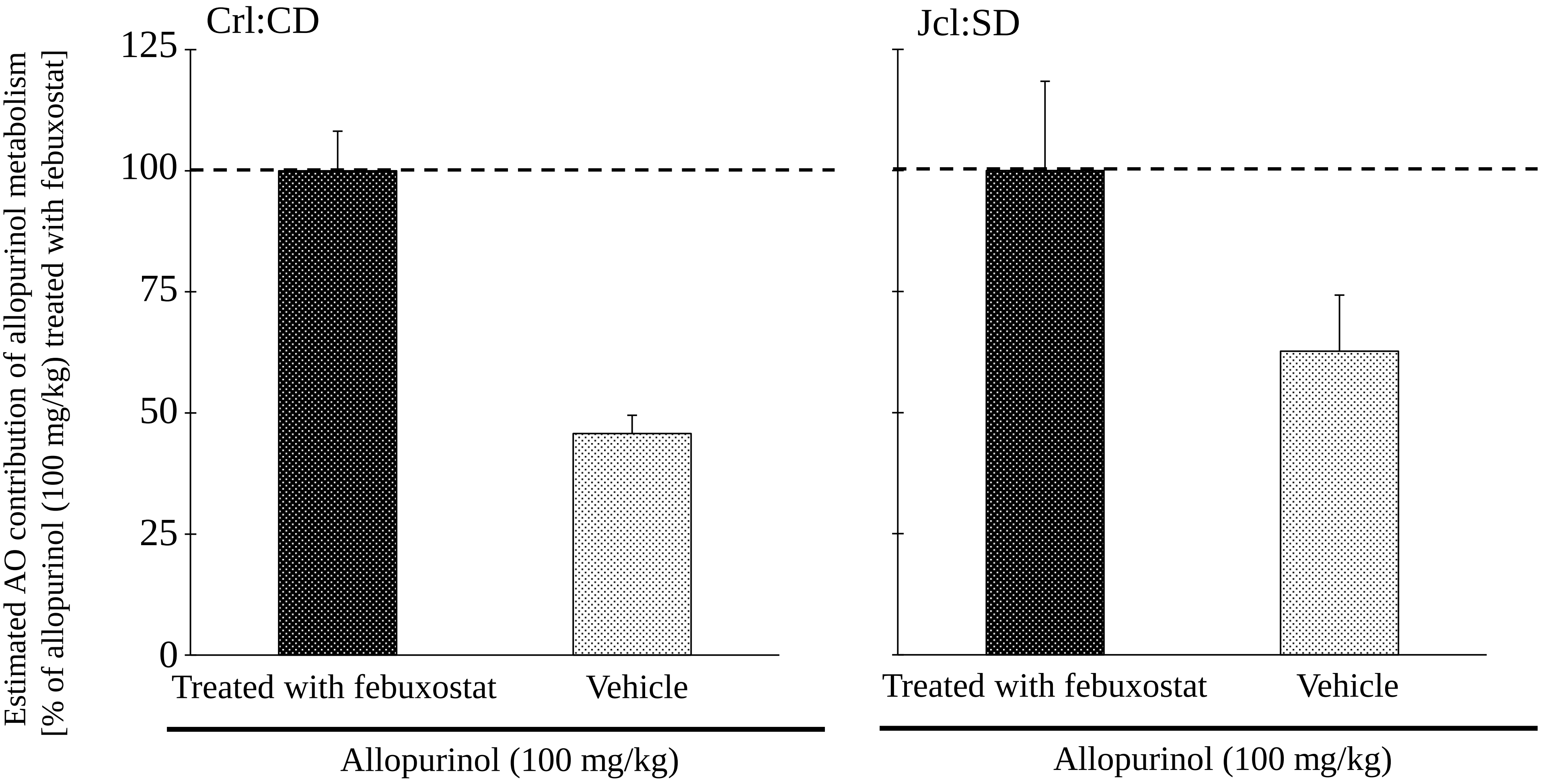

Supplemental Fig. 2

Estimated AO contribution of allopurinol metabolism
considering complete inactivation of XO with Allopurinol (100 mg) and febuxostat treatment.
